# htseq-clip: a toolset for the preprocessing of eCLIP/iCLIP datasets

**DOI:** 10.1093/bioinformatics/btac747

**Published:** 2022-11-17

**Authors:** Sudeep Sahadevan, Thileepan Sekaran, Nadia Ashaf, Marko Fritz, Matthias W Hentze, Wolfgang Huber, Thomas Schwarzl

**Affiliations:** Genome Biology / Directors' Research, European Molecular Biology Laboratory (EMBL), Heidelberg 69117, Germany; Genome Biology / Directors' Research, European Molecular Biology Laboratory (EMBL), Heidelberg 69117, Germany; Directors' Research, Previously European Molecular Biology Laboratory (EMBL), Heidelberg 69117, Germany; Directors' Research, Previously European Molecular Biology Laboratory (EMBL), Heidelberg 69117, Germany; Genome Biology / Directors' Research, European Molecular Biology Laboratory (EMBL), Heidelberg 69117, Germany; Genome Biology / Directors' Research, European Molecular Biology Laboratory (EMBL), Heidelberg 69117, Germany; Genome Biology / Directors' Research, European Molecular Biology Laboratory (EMBL), Heidelberg 69117, Germany

## Abstract

**Summary:**

Transcriptome-wide detection of binding sites of RNA-binding proteins is achieved using Individual-nucleotide crosslinking and immunoprecipitation (iCLIP) and its derivative enhanced CLIP (eCLIP) sequencing methods. Here, we introduce htseq-clip, a python package developed for preprocessing, extracting and summarizing crosslink site counts from i/eCLIP experimental data. The package delivers crosslink site count matrices along with other metrics, which can be directly used for filtering and downstream analyses such as the identification of differential binding sites.

**Availability and implementation:**

The Python package htseq-clip is available via pypi (python package index), bioconda and the Galaxy Tool Shed under the open source MIT License. The code is hosted at https://github.com/EMBL-Hentze-group/htseq-clip and documentation is available under https://htseq-clip.readthedocs.io/en/latest.

## 1 Introduction

In recent years, a 4-figure number of novel RNA-binding proteins (RBPs) was discovered, many of them containing disease-associated mutations (Gebauer *et al.*, 2021). Crosslinking and immunoprecipitation (CLIP) coupled with next-generation sequencing (NGS) is the current state of the art used for the transcriptome-wide detection of RBP binding sites to study their underlying mechanisms. Individual-nucleotide CLIP (iCLIP) (Konig *et al.*, 2011) methods and its derivatives, enhanced CLIP (eCLIP) (Van Nostrand *et al.*, 2016), infrared-CLIP (irCLIP) (Zarnegar *et al.*, 2016), easyCLIP (Porter *et al.*, 2021) and others use UV light to induce a covalent bond between the protein and the bound RNA. The property of reverse transcriptase enzymes to stop heuristically at those crosslinks results in truncations. Occasional skipping of these sites can introduce mutations or deletions. A handful of tools such as PIPE-CLIP and pyCRAC have been developed for the preprocessing of CLIP datasets. PIPE-CLIP was designed primarily for processing HITS-CLIP, PAR-CLIP and iCLIP datasets (Chen *et al.*, 2014). pyCRAC is mainly aimed at the processing of CLIP and CRAC (crosslinking and cDNA analysis) datasets (Webb *et al.*, 2014). Our recently developed R/Bioconductor package DEWSeq uses a sliding window approach based on DESeq2 (Love *et al.*, 2014) for the analysis of CLIP datasets. For this, the data need to be prepared as follows: truncation sites need to be extracted as individual nucleotide positions and summarized into sliding windows, which are generated based on a user provided gene annotation file in gff3 format.

Here, we present *htseq-clip*, an open-source Python package, for this type of pre-processing of CLIP datasets. *htseq-clip* can be used to extract truncation, mutation or deletion sites from CLIP NGS data and to summarize these data into a count matrix format for downstream analyses. In addition, it provides metrics such as crosslink site density and maximum number of crosslink sites for an individual nucleotide for filtering.

## 2 Design and implementation

The development of *htseq-clip* was inspired by popular bioinformatics tools such as htseq-count (Anders *et al.*, 2015), bedtools (Quinlan and Hall, 2010) and featureCounts (Liao *et al.*, 2014). Although these software packages can be used in combination, for processing and counting crosslink sites from i/eCLIP datasets, *htseq-clip* simplifies the process and provides additional statistics for crosslink sites and annotation features.

*htseq-clip* is designed to extract crosslink sites from a wide range of CLIP protocols. Based on user-specified input parameters, *htseq-clip* can extract truncation sites with an offset from either the 5′ end or the 3′ end of the read, from either the first mate or the second mate of an alignment through command line parameters. Furthermore, htseq-clip is able to aggregate these sites either into entire gene features (intron, exon, UTR) or into sliding windows over these features. Thus, htseq-clip allows a user to have granular control over the information extracted from i/eCLIP datasets.

*htseq-clip* is written in Python 3 and builds upon the *htseq* package (Anders *et al.*, 2015; Putri *et al.*, 2022). *htseq* includes parsers for the most commonly used file formats in genomics.

### 2.1 System requirements

The system requirements of htseq-clip are the same as those of *htseq*, namely Linux or macOS.

### 2.2 Data requirements

*htseq-clip* requires the following inputs:

1. A gene annotation file in .gff3 format, as it is available from the GENCODE project for example.
2. Alignment (.bam) files after polymerase chain reaction (PCR) duplicate removal using unique molecular identifiers (UMIs). Ideally, the experiment should be performed in replicates with proper size-matched input controls.

### 2.3 Data processing workflow

The workflow implemented in *htseq-clip* can be grouped into three steps:

1. Prepare annotation: flatten the gene annotations to unique annotations for chromosomal positions, create sliding windows from flattened annotation and a mapping file for downstream analyses.
2. Extract crosslink sites: extract truncation, deletion, insertion sites and summarize them as counts per sliding window.
3. Count and summarize crosslink sites: summarize counts of multiple samples to a count matrix for further analysis.

**Fig. 1.**
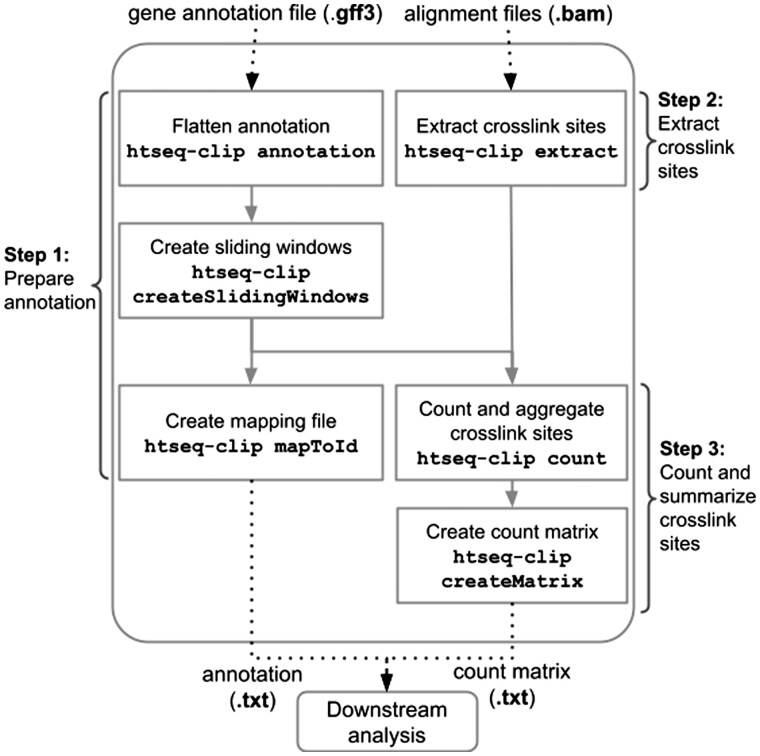
htseq-clip data processing workflow

Figure 1 illustrates the data flow and commands used in processing CLIP datasets with htseq-clip.

#### 2.3.1 Prepare annotation

The first step in processing i/eCLIP data using htseq-clip is to prepare the annotation file. This step requires a .gff3 formatted gene annotation file as input. The supplied annotation file is flattened into BED6 format and is further used to create sliding windows. Users can set the width and step (slide) of the windows. The gene feature boundaries are taken into consideration at this step such that the sliding windows do not span multiple gene features (e.g. a window beginning in an exon of a gene and ending in the neighboring intron). The last step is to generate a mapping file. This file contains all the metadata related to a sliding window such as: window id, window size, step size, corresponding gene, gene feature, gene type, gene name and gene id. This information is essential for downstream filtering, quality control and analyses.

#### 2.3.2 Extract crosslink sites

In this step, alignment files are processed and crosslink sites are extracted in BED6 format. The parameters used in this step give the user flexibility to define the read/mate, crosslink type and offset length parameters which can be used to convert an RT stop position into a crosslink site position. Additionally, users also have the flexibility to choose alignment quality, minimum and maximum lengths of reads and whether only primary alignments or both primary and secondary alignments of a read can be used for crosslink site counting. An additional advantage of extracting crosslink sites in BED6 format is that users gain flexibility to convert the crosslink site file into bigWig or bedGraph format, for visualization using a genome browser.

#### 2.3.3 Count and summarize crosslink sites

In this step, a sliding window file created in the first step is used to count and summarize the crosslink sites into sliding windows. Along with the total number of sites per window, additional information such as length of the window, number of positions in a window with crosslink sites, maximum site count in the window and crosslink site position density (ratio of number of nucleotides with crosslink sites compared to the length of the window) are also given. In downstream analysis, users may filter the sliding windows based on this information. As an additional step, crosslink site counts from multiple samples can be concatenated into one matrix, creating a site count matrix file. This step is intended to create an R-friendly output matrix file, as R/Bioconductor packages are the default choice for statistical analysis in bioinformatics pipelines.

## 3 Summary

*htseq-clip* provides an easy-to-use solution for the pre-processing of i/eCLIP alignment files using custom gene annotations. The parameter combinations available in *htseq-clip* enable users to work with data from a wide variety of common CLIP protocols. The output crosslink site matrix and other metrics can be used for downstream analysis such as the R/Bioconductor Package *DEWSeq*.

## Data Availability

There are no new data associated with this article.
